# Utilising massive open online courses to enhance global learning dissemination in cleft lip and palate: a case report of penta helix collaboration

**DOI:** 10.1186/s12909-024-05225-4

**Published:** 2024-03-18

**Authors:** Erli Sarilita, Anggun Rafisa, Priya Desai, Peter A. Mossey

**Affiliations:** 1 Department of Oral Biology, Faculty of Dentistry, Universitas Padjadjaran, Bandung, Indonesia; 2 Research and Innovation, Smile Train, New York, USA; 3 Division of Oral Health Sciences and WHO Collaborating Centre for Oral Health & Craniofacial Anomalies, University of Dundee, Dundee, Scotland UK

**Keywords:** MOOC, Case report, Orofacial cleft, COVID-19, Online learning

## Abstract

**Background:**

Educating and raising awareness in cleft lip and palate future generations is one vital effort to ensure the improvement of cleft care and research in the future. This study reported the overview in organising and evaluating the Massive Open Online Course (MOOC) in Cleft Lip and Palate as the alternative way for students’ capacity building outside their study program whilst also earning credits towards their studies.

**Methods:**

Smile Train cleft charity generously donated recorded lectures from cleft experts around the world in which each of the experts agreed to provide one-hour live discussion sessions. The learning activities ranging from lectures, pre- and post-course evaluation, forum, live discussion sessions, virtual visits to Indonesian Cleft Centre, self-reflection assignments and final project. A survey was released to the participants to collect their feedback.

**Results:**

The course mainly attracted dental students, and several allied health professional students. In total, 414 out of 717 participants registered for this MOOC managed to finish the course and received a certificate of completion which was run between August–October 2021. In general, participants positively received the course.

**Conclusions:**

The MOOC model and its objective of disseminating widespread information across geographical boundaries to enhance learning about cleft lip and palate treatment was achieved. This report serves as an example for other educational institutions and stakeholders who plan to use online educational engagement platforms to provide high-quality education and capacity building to participants in lower-middle income countries.

**Supplementary Information:**

The online version contains supplementary material available at 10.1186/s12909-024-05225-4.

## Introduction

Cleft lip and or palate (CLP) is a congenital malformation, which can be either syndromic or non-syndromic, and results in disturbances in feeding, swallowing, speech, and esthetics, and may lead to child mortality if left untreated [1]. The treatment itself requires a multidisciplinary approach, and sometimes, depending on the severity, may require multiple surgeries and longitudinal care [1]. Barriers to cleft care are aggravated by difficulties in access to care, lack of family awareness and inadequacy of experienced healthcare professionals related to cleft [2].

Only a few papers explored the educational aspects of cleft care and all were focusing on surgical skill improvement through 3d graphics software and internet-based simulation [3–5]. Cleft care requires comprehensive and multidisciplinary treatments which involve beyond surgery. Previous study found that the awareness of college students in cleft treatment was considerably low [6]. Looking to the future, it is necessary to invest in the education of the young generation to secure sustainable cleft care.

The global COVID-19 pandemic disrupted the education field worldwide in 2020. However, higher education has made strides to adapt to the COVID-19 challenges and to innovate the learning activities through online or hybrid learning [7]. Although e-learning or Massive Open Online Courses (MOOC) seems to have become the appropriate answer in tackling distance education, especially during special circumstances such as COVID-19 pandemic, it also has some difficulties. The challenges faced by Indonesian students in joining an internet-based education included disparity of adequate internet resources in cities and rural areas, combined with the 3 time zones across the country. The aim of a MOOC in this context is the provision of an educational platform accessible by those in low resource settings, targeting the problems of inequality in access to care and invisibility in access to data and facilitating essential data collection. This also brings in awareness of the sustainable development goals (SDGs) and the need for universal health coverage (UHC) in low resource settings.

In 2021, during a challenging pandemic period, a MOOC on cleft lip and palate treatment was held by the Universitas Padjadjaran in collaboration with relevant prominent stakeholders involving international experts, hosting domestic and foreign participants. This paper serves as a case study for the first MOOC on cleft lip and palate, and described the perceived study outcomes for further consideration in creating similar MOOC.

## Methods

The Ministry of Education, Culture, Research and Technology (MoECRT) of the Republic of Indonesia released an open call for the Micro-credential for Undergraduate Students in Indonesia (KMMI) grant program to all higher education institutions in Indonesia in May 2021 during the COVID-19 pandemic. The educational mission of this initiative was to break the boundaries of Indonesian undergraduate students to study anywhere they need. This grant allowed the appointed higher education to organise a free of charge, online 8-week course which was run between August to October 2021, in a topic agreed by MoECRT, with study outcomes transferable to the student’s academic transcript equals to 3 sks (credits in Indonesian higher education system). The course was designed to operate in collaboration with industry, government or NGOs, and was offered to undergraduate students all over Indonesia for a certain participants quota set by MoECRT Universitas Padjadjaran, in which the selected students receive some living allowances from MoECRT.

The Universitas Padjadjaran immediately set up an organisational team of academics to arrange a proposal to the MoECRT. The first author (ES) was appointed as the project leader of the central MOOC-KMMI Universitas Padjadjaran committee following her experience in attending an MOOC organised by University of Dundee during her education there via FutureLearn platform. The central team decided that the Universitas Padjadjaran owned Moodle-based MOOC platform could be utilised for this program (https://mooc.live.unpad.ac.id/).

Within a month, the team managed to gather prominent stakeholders and set several MOOC titles relevant to the available stakeholders, complete with the course syllabus ready for submission to the MoECRT (Additional file 1). Specifically for the MOOC on cleft lip and palate treatment, we partnered with Smile Train and the Indonesian Cleft Centre (YPPCBL). Smile Train is the largest cleft charity in the world. YPPCBL is the oldest cleft centre in Indonesia with over 42 years experiences caring for cleft patients, working hand in hand on a daily basis with the Universitas Padjadjaran, which is located within the complex of Faculty of Dentistry and Dental Teaching Hospital of the Universitas Padjadjaran. MoECRT announced that the Universitas Padjadjaran was one of the MOOC-KMMI grant winners to host 1480 undergraduate students in 6 MOOC courses, one of which was MOOC cleft lip and palate welcoming 400 KMMI participants.

After the award announcement, the organising committee was expanded by involving administrative staff and also students as organising committee. The first author (ES) was also chair of the MOOC cleft lip and palate managerials. The communication between the organising committee, Smile Train, and YPPCBL was intensified. YPPCBL, ES and PM discussed the preferable modules. ES, PM and Smile Train formulating the possible international experts to teach in this MOOC. With the advocacy of Smile Train, the current MOOC on cleft lip and palate treatment received existing recorded lectures from West African College of Surgeons (WACS).

After carefully designing the contents of the course and its flow, Smile Train continued their advocacy role in corresponding to the respective experts regarding their schedule for synchronous online discussion. The student organising committee which was a committee of Transformative Summer Elective Course in Dentistry (Transcendent) 2021 were trained on how to manage the MOOC platform for 2 weeks and pilot test the courses.

The MOOC was run between August–October 2021 in a predefined course structure (Additional file 1). All the case study protocols in this study including the data collection, analysis, and reports were ethically exempted by the Research Ethics Committee Universitas Padjadjaran (No.1246/UN6.KEP/EC/2022). The number of participants enrolled in the course and the number of course completers were recorded. The demography of the learners was also recorded at the beginning of the course.

A set of post-course questionnaires designed by experts in Smile Train were embedded at the end of the MOOC as a mandatory phase before the certificate of completion request (Additional file 2). Therefore, the questionnaires were returned only by completed users. The data collection stage underwent a validation process through pilot testing with a small sample of respondents who are both participants and student committee members. This process helped identify any ambiguities, confusing questions, or other issues with the survey instrument.

The questionnaire was designed to evaluate the course structure, outcome, and general information. Section 1 was designed to understand how future courses may need to be adjusted to best respond to students’ needs. Participants were asked to respond using a 5-point Likert scale ranging from 1 to 5 where 1 strongly disagree and 5 strongly agree. Four of the questions in section one which was concerned with course content and quality were open-ended. Section 2 regarding outcome evaluation was intended to observe how effective the course was in teaching students the desired material. The first six questions of section 2 compared the knowledge of the learners before and after course. Three questions after that were multiple choice. At the end of the second section, the questionnaire asked participants to complete one open-ended question. Stacked bar charts were used to depict results of Likert analysis. Open-ended questions were processed using an open-source Word Cloud Generator to identify the most frequently mentioned themes that emerged from the responses (https://monkeylearn.com/word-cloud.).

Promoting the MOOC-KMMI Universitas Padjadjaran was performed using the media channels of both MoECRT and Universitas Padjadjaran (Instagram and website). In addition, we applied the current trend of younger generation social media engagement on TikTok by one lecturer to gather more attention from the undergraduates. Smile Train also disseminated the information through their well-established network. MOOC-KMMI was targeting undergraduate students in which the registration can be found in the KMMI MoECRT official website (https://kmmi.kemdikbud.go.id/).

Five hundred and 99 undergraduate students applied to the MOOC cleft lip and palate to the KMMI MoECRT website. The organising committee performed selection to reduce the participants to 400 as per the quota set by MoECRT based on the relevancy of the educational background. The final list of participants was exported from the KMMI MoECRT dashboard and then enrolled automatically to the MOOC platform (https://mooc.live.unpad.ac.id/). Nevertheless, the MOOC also received a large number of registrations from people who are not suitable for the MOOC-KMMI criteria such as postgraduate students and clinical dental students from both Indonesia and abroad. The Indonesian dental education program is slightly different from other countries, in that students must pursue 4 years of Bachelor of Dental Science (undergraduate; pre-clinical) and then continue to 2 years of professional dentist degree (postgraduate; clinical). Considering the mission was to ensure the sustainability of cleft care by educating the younger generations, we decided to open an MOOC for non-KMMI routes, where all learning activities were the same except the non-KMMI route participants did not receive a stipend from the MoECRT. Therefore, in total, 717 participants registered to the MOOC platform and enrolled to the course, in which 400 were KMMI MoECRT funded undergraduate students and 317 were non-KMMI MoECRT (regular participants).

The MOOC was managed and ran for 8 weeks, in which each week there were live/synchronous sessions for meeting the expert using the Zoom meeting platform. Some rescheduling from the initial plan happened several times which was normal during the pandemic era. Drop out participation was also noted as typical in MOOC type of learning.

To evaluate the MOOC, a specific set of questionnaires for post-course feedback was generously designed by Smile Train. The course quality can be reflected in the level of satisfaction expressed by participants in their feedback [8]. The participants were required to fill the survey as a mandatory step to receive an e-certificate of completion with grades. Reports have been received where the MOOC certificates were successfully transferred to the students’ academic transcripts. The demographic survey of the participants and the post-course survey were analysed using descriptive statistics.

## Results

**Fig. 1:**
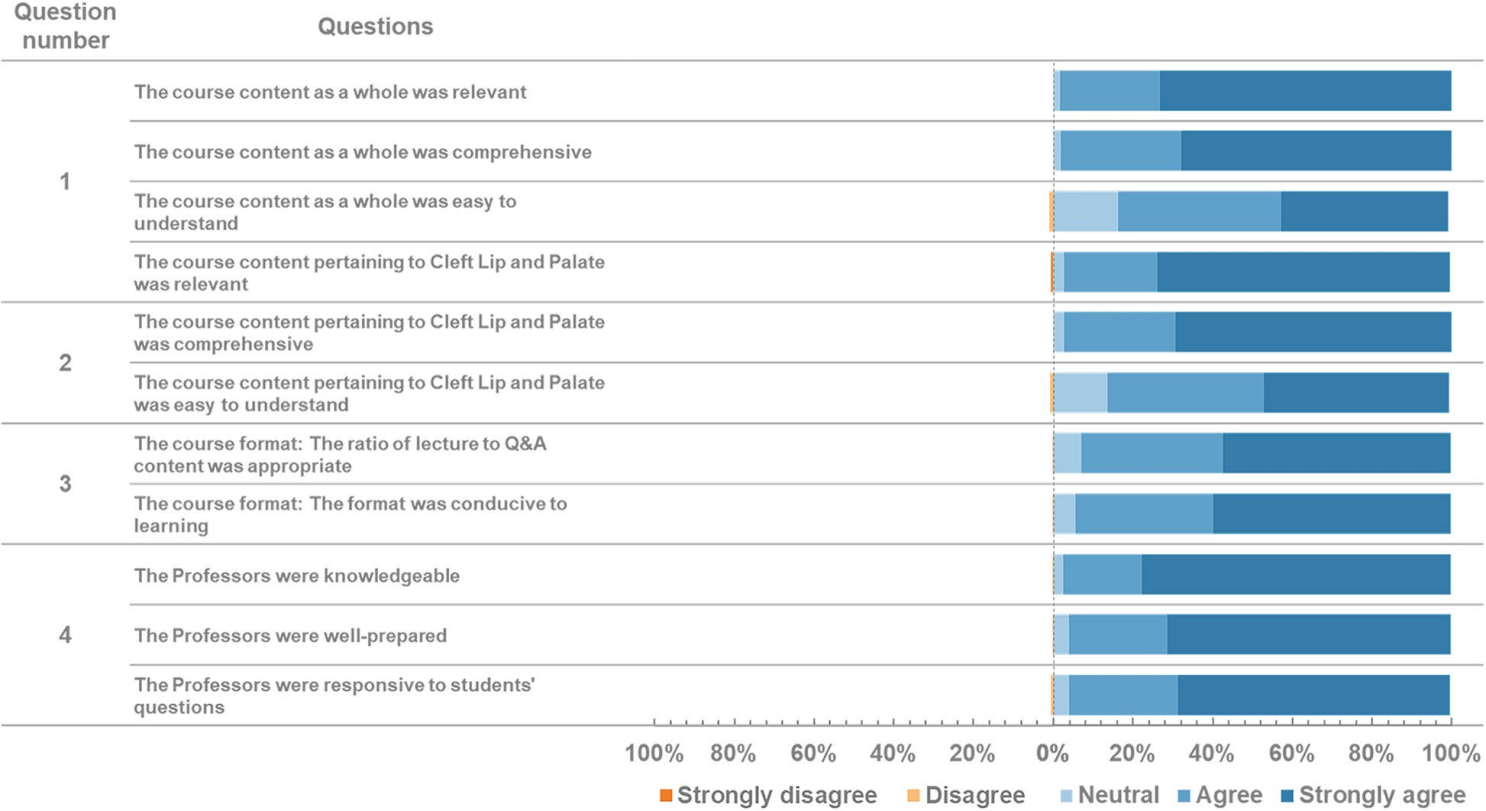
The results of the post-course survey. Section I: Course Evaluation, questions 1–4

**Fig. 2:**
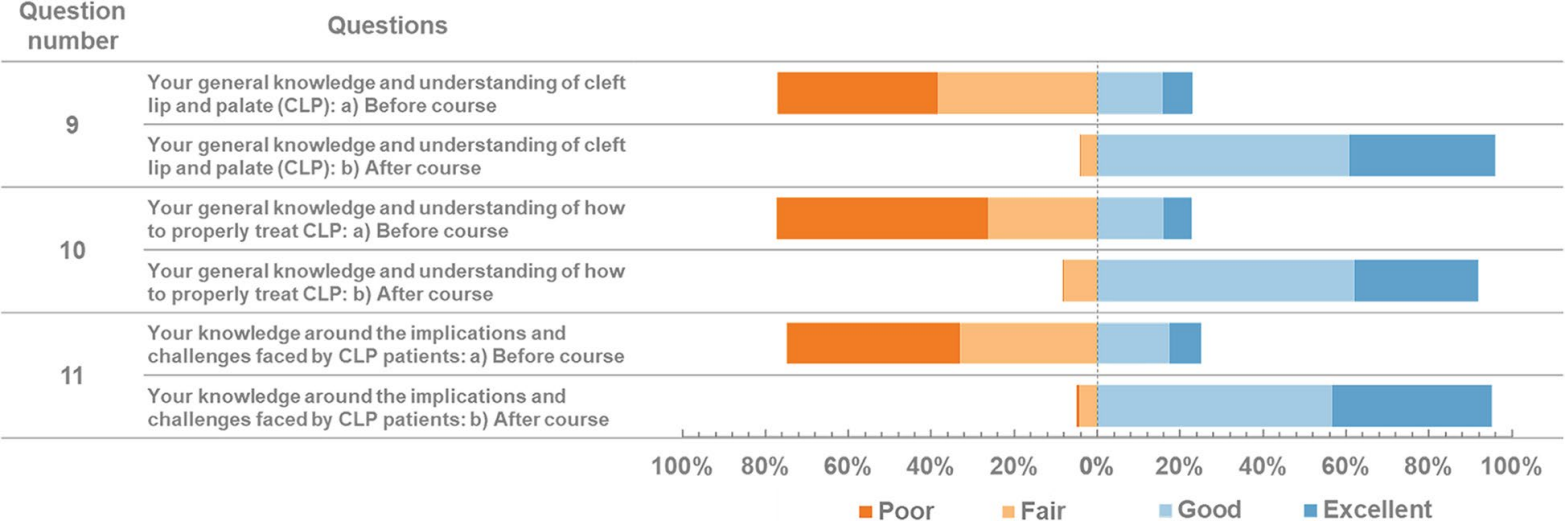
The results of the post-course survey. Section II: Outcome Evaluation, questions 9–11

First of all, the registered participants were observed through the respondents’ characteristics. From 717 registered participants to the MOOC platform, 625 (87.17%) were Indonesian and the rest (*n* = 92) were from outside Indonesia (Australia, Cambodia, Ghana, India, Indonesia, Japan, Malaysia, Myanmar, Nepal, Netherlands, Nigeria, Pakistan, Singapore, Vietnam) (see Table 1). A majority of the participants were students (undergraduate *n* = 606; master program *n* = 8; clinical master program *n* = 80; doctoral program *n* = 9) and the rest were non-students composed of general dentists and oral maxillofacial specialised dentists (*n* = 14). Out of the total of 717 participants who registered, only 414 successfully finished the course. See Fig. 1, Table 2, Fig. 2, and Table 3 for details on the results of the post-course survey.

**Table 1:** Profiles of the course completers

**Country**	**#participants**
Indonesia	391
Malaysia	12
Nigeria	6
Cambodia	1
Myanmar	2
Pakistan	1
Vietnam	1
Grand Total	414
**Area of specialty**	**#participants**
Dentistry	308
Medical	43
Nursing	41
Psychology	4
Public Health	4
Social work	1
Speech pathology / related	2
Biomedical engineering	5
Dental therapy	2
Nutrition	2
Physiotherapy	1
Health Technology	1
Grand Total	414

**Table 2:** The results of the post-course survey. Section I: course evaluation, questions 5–8

Question number	Questions	Top three responses
5	What did you like most about this course, and why?	• Expert professors • Easy to understand • Flexible time
6	What did you like least about this course, and why?	• Slow internet connection • Advanced topics • Clashing schedule
7	Please provide up to three examples of how you will apply the learnings from this course.	•Share awareness of CLP to others • Take further study degree to treat CLP patients • Apply healthy lifestyle to prevent CLP
8	Would you recommend this course to someone else?	99% Yes

**Table 3:** The results of the post-course survey. Section II: outcome evaluation, questions 12–14

Question number	Questions	Top three responses
12	What aspect of treating CLP did you learn the most about?	• Multidisciplinary Team Approach to Cleft Care • Nutritional Therapy • Genetics in Cleft
13	From the topics of the course, which topic is your most favourite and would like to learn more about?	• Rhinoplasty, Labioplasty, Palatoplasty, Velopharyngealplasty • Telemedicine in Cleft Care • Multidisciplinary Team Approach to Cleft Care • Nutritional Therapy
14	From the lectures offered, which topic is the easiest and most understandable?	• Aetiology of Orofacial Cleft • Telemedicine in Cleft Care • Nutritional Therapy

The demographics of the completed participants were mainly from Indonesia with small contributions from several countries in Asia and Africa. The educational background of the completed participants were dental, medical and nursing students contributing to 84 participants, and the rest were from allied health professionals (see Table 1).

The post-course survey revealed a positive agreement towards the relevance and comprehensiveness of the course. In addition, the majority of the participants agreed that the course was easy to understand, lecture-discussion ratio was appropriate, and the course format enhanced the learning process. The tutors were also given positive feedback regarding their knowledge, preparedness, and responsiveness to the student’s questions (see Fig. 1).

Multidisciplinary Team Approach to Cleft Care and Nutritional Therapy were the two topics which were chosen by the participants for being most learned about. Rhinoplasty, Labioplasty, Palatoplasty, Velopharyngealplasty; and Telemedicine in Cleft Care were the top two most favourite and raising curiosity from the participants. Aetiology of Orofacial Cleft received the most votes for being the easiest and most understandable topic (see Table 3).

## Discussion

The current development of MOOC on cleft lip and palate followed previous studies regarding 10 simple rules in MOOC production which were determining the educational mission, experiencing a MOOC, choosing the platform, deciding a topic, arranging the MOOC team personnel, designing the MOOC contents, testing and then promoting the MOOC, executing the MOOC, and finally evaluating the MOOC [9].

Following comprehensive discussion and weighing all the available resources, the MOOC cleft lip and palate was decided to be composed of lectures (both live - synchronous and recorded - asynchronous), meet the expert (both live - synchronous and recorded - asynchronous), asynchronous quiz pre and post each module, and reflective presentation after each week. The combination of the advantages of both synchronous and asynchronous learning materials were deemed important in this course. Synchronous learning allowed for real-time interaction with experts and immediate feedback. Asynchronous learning provided flexible scheduling, reducing time constraints during the learning process and allowing for a self-paced experience. Each module was set in sequence, in which a participant cannot continue to the next module before completing all the necessary learning activities. The quality check method employed in this MOOC was user feedback and testing. Before the release of each learning step in the MOOC, testing was conducted with a small group of participants to identify and address any technical errors. YPPCBL played a vital role in providing their cleft centre and patients as the setting of the virtual visiting short documentary.

The course recorded a 42.2% drop out rate, whereas 414 out of 717 registered participants obtained a certificate of completion. This dropout rate is considerably low compared to previous study reports on MOOC which stood approximately 90% [10, 11]. This questionnaire-based study relied on course completers, which should not undermine the validity of the research.

This case study of a successful launch of an MOOC cleft lip and palate, alongside previous MOOCs in other fields that had comparable positive outcomes [12–16], can offer some guidance to higher education institutions considering similar MOOC-based programs. One major reason students reported persevering through questionnaires was that the learning experience was perceived as being easy to understand. In addition, knowledgeable professors and flexibility were also mentioned frequently.

Despite these promising results, based on several participants’ feedback, unfavourable aspects of the MOOC were difficulties in learning due to internet accessibility, clashing schedules, and the topics that are more advanced than they expected. Therefore, some aspects of the MOOC required revision for better performance in the future such as refining the syllabus appropriate for the expected targeted participants and covering one domain of cleft care at a time rather than pooled all facets of cleft care in one MOOC. However, clashing schedules and internet connection were out of the course organisers scope to manage. Due to geographical location, time differences were unavoidable, and matching all participants’ schedules was difficult. Moreover, questionnaires specifically targeted to evaluate MOOC should be designed and tested prior to utilisation. Furthermore, formative assessment was not conducted in this MOOC due to time constraints in course planning, which remains a limitation of this COVID situation-derived project. This MOOC marked the first step into the realm of distance education in the cleft lip and palate field, offering a promising starting point for future enhancements in delivery and research. Drawing from the insights gained in this study, it becomes essential to develop courses that emphasize learner engagement and strive for optimal effectiveness.

The increasing awareness of younger generations of cleft patients has certainly been visible considering the most frequent answers to the follow up action post course were to share awareness of CLP to others, take further study degrees to treat CLP patients, and apply healthy lifestyle to prevent CLP. Some students’ self-reflection assignments of the MOOC were seen after typing “mooc cleft” in the web search engine. The young participants also noted that they are now realising the multidisciplinary treatments should be borne by children with clefts.

This study, along with a similar MOOC case study in Indonesia, demonstrated that the pandemic did not hinder global learning for allied health students or clinicians, and that MOOCs are feasible in limited-resource settings [17]. The primary effect was observed in the sudden increase of allied health students’ engagement in online learning. The government played a significant role in providing financial support for academics to gather all the necessary resources to maintain the quality of global learning activities. This precedent has also provided academics in Indonesia with the necessary and sustainable learning technology skills that will be beneficial in the long term, even after the pandemic is over. In addition, delivering a course via an MOOC offers benefits such as scalability, cost-effectiveness, flexibility, global accessibility, and specialised topics. These advantages go beyond addressing COVID-related challenges and support lifelong learning and continuous improvement.

**Fig. 3:**
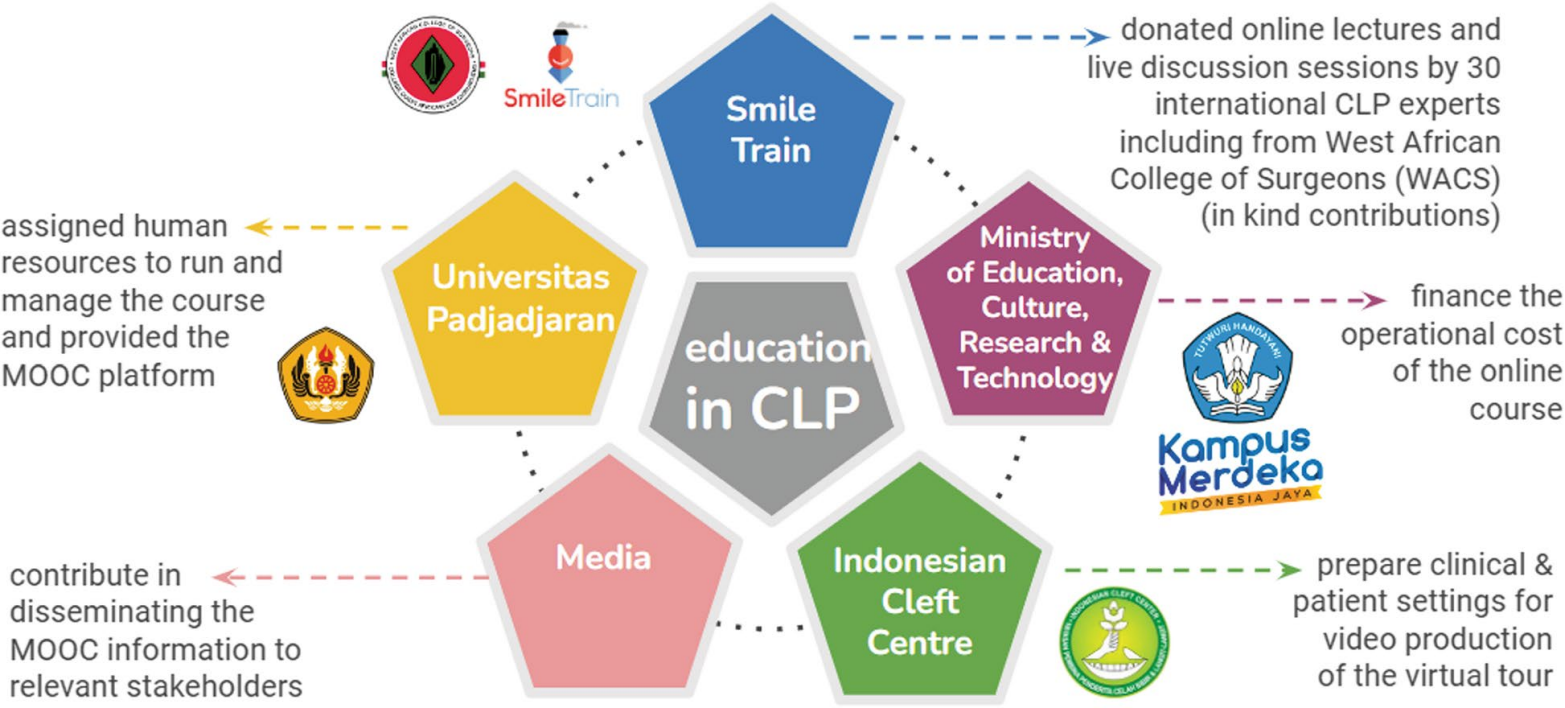
Penta helix collaboration in MOOC cleft lip and palate

The Indonesian government has engaged with and financially supported this innovative collaborative learning MOOC program concerned with sustainable education through capacity building on the topic of comprehensive cleft care through the MOOC-KMMI grant to Universitas Padjadjaran in 2021. In addition, Smile Train and YPPCBL pledged effort in cleft education was remarkable, in which without their contribution, this MOOC cleft lip and palate would not have taken place. This MOOC is proof that strong penta helix collaboration between a higher education institution, government, industries/NGOs, community, and media can yield such an impact in cleft care (Fig. 3). This partnership has brought together international experts and students who were initially separated by a combination of geographical distance, time difference and pandemic. This MOOC grant produced multiplier effects in increasing the skill of one higher institution in managing a challenging new project amid pandemic while also gaining internationalisation perspectives in terms of inviting international lectures and participants. Not to mention, new international networks introduced by Smile Train will foster research partnerships in the long run.

## Conclusion

MOOC has proven to be an effective tool in breaking geographical boundaries, gathering students and lecturers from all over the world in one platform. Participants’ feedback was positive. A collateral benefit is the building of a cross regional research network for future collaborations in the field, and serves as a model for stakeholders who plan to enhance both education and research in order to provide better quality of care in a particular field, promote capacity building using innovative technologies in education, and make a contribution to UHC.

### Supplementary Information

**Supplementary Material 1.**

**Supplementary Material 2.**

## Data Availability

The datasets generated and/or analysed during the current study are not publicly available due to respondents’ confidentiality but are available from the corresponding author on reasonable request.

## Abbreviations

MOOC: Massive open online course
CLP: Cleft lip and or palate
SDGs: Sustainable development goals
UHC: Universal health coverage
MoECRT: Ministry of education, culture, research and technology
KMMI: Micro-credential for undergraduate students in indonesia
WACS: West african college of surgeons
Transcendent: Transformative summer elective course in dentistry
