# Supplementary material for: Utilising massive open online courses to enhance global learning dissemination in cleft lip and palate: a case report of penta helix collaboration
Source: BMC Med Educ. 2024 Mar 18;24:301. doi: 10.1186/s12909-024-05225-4 (PMC10949738; doi:10.1186/s12909-024-05225-4)
Supplement: Supplementary file 1 — Supplementary Material 1. [file 12909_2024_5225_MOESM1_ESM.docx]

## Additional file 1. MOOC cleft lip and palate structure and syllabus

##
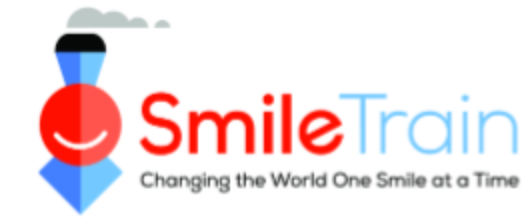


##
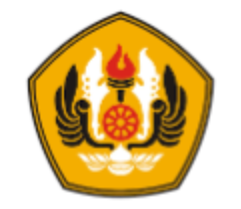

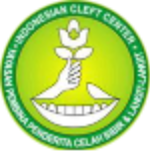

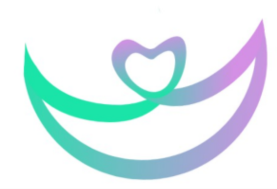

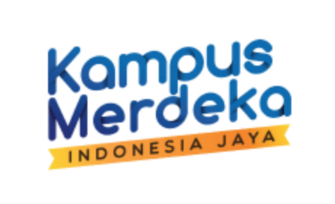
KMMI Course Syllabus MOOC Cleft Lip & Palate

| **Month** | **Week** | **Learning outcomes** | **Topics** | **Method and Media** | **Instructional strategies** | **Assessments** | | **Time (hour)** |
| --- | --- | --- | --- | --- | --- | --- | --- | --- |
|  |  |  |  |  |  | **Indicators** | **Techniques** |  |
| I | 1 | To identify the aetiological principles of cleft lip and palate from the environmental and genetic aspect | Introduction to Cleft Lip and Cleft Palate | Asynchronous (MOOC); Synchronous (ZOOM) | T: Lectures & self-learning  P: Live Discussion with University Professors and Smile Train Experts  AA: Pre-test, post-test | Basic understanding of cleft lip and palate aetiology; the role of environmental factors in cleft lip and palate; the developmental processes of cleft lip and palate in the fetus; the genetic aspects of cleft lip and palate | Assignment: Self-assessment for this week's learning activities  Post-test for each study material: Multiple choice questions | 18 |
|  |  |  | Aetiology of Orofacial Clefts, The Role of Environmental Factors |  |  |  |  |  |
|  |  |  | Developmental Anatomy of Cleft Lip and Cleft Palate |  |  |  |  |  |
|  |  |  | Genetics in Cleft 1 |  |  |  |  |  |
|  |  |  | Genetics in Cleft 2 |  |  |  |  |  |
|  | 2 | To identify the basic principles of pre-surgical nasoalveolar molding | Pre-surgical Nasoalveolar Molding | Asynchronous (MOOC); Synchronous (ZOOM) | T: Lectures & self-learning  P: Live Discussion with University Professors and Smile Train Experts  AA: Pre-test, post-test | Basic understanding of pre-surgical nasoalveolar molding; Basic understanding of nasal reconstruction surgery in cleft lip and palate | Assignment: Self-assessment for this week's learning activities  Post-test for each study material: Multiple choice questions | 18 |
|  |  |  | Nasoalveolar Molding for Unilateral and Bilateral Cleft Lip and Palate: Clinical Steps |  |  |  |  |  |
|  |  | To identify the basic surgical principles of nasal reconstruction in cleft lip and palate | Rhinoplasty |  |  |  |  |  |
|  |  |  | Cleft Rhinoplasty/ Nose Revision Surgery |  |  |  |  |  |
|  | 3 | To identify the basic principles of cleft lip reconstruction surgery | Bilateral Cleft Lip Repair | Asynchronous (MOOC); Synchronous (ZOOM) | T: Lectures & self-learning  P: Live Discussion with University Professors and Smile Train Experts  AA: Pre-test, post-test | Basic understanding of cleft lip reconstruction surgery; Basic understanding of cleft palate reconstruction surgery | Assignment: Self-assessment for this week's learning activities  Post-test for each study material: Multiple choice questions | 18 |
|  |  |  | Unilateral Cleft Lip Repair |  |  |  |  |  |
|  |  | To identify the basic principles of cleft palate reconstruction surgery | Palatoplasty |  |  |  |  |  |
|  |  |  | Cleft Palate Repair |  |  |  |  |  |
|  |  |  | Cleft Palate |  |  |  |  |  |
|  | 4 | To identify the basic principles of alveolar bone graft in cleft lip and palate | Alveolar Bone Graft | Asynchronous (MOOC); Synchronous (ZOOM) | T: Lectures & self-learning  P: Live Discussion with University Professors and Smile Train Experts  AA: Pre-test, post-test | Basic understanding of alveolar bone grafting; Basic understanding of anaesthetic principles and its complications in cleft lip and palate surgery; Basic understanding of post-operative treatment | Post-test for each study material: Multiple Choice Questions | 18 |
|  |  | To identify the basic anaesthetic principles and its complication in the cleft lip and palate surgery | Cleft Anaesthesia |  |  |  |  |  |
|  |  |  | Common Anaesthetic Complications Arising from Cleft Surgery |  |  |  |  |  |
|  |  | To identify the basic principles of post-operative treatment | Post-Op Nursing Care of Cleft Patients |  |  |  |  |  |
| II | 1 | To acknowledge the challenges of cleft lip and palate surgical treatment in low middle-income countries during COVID-19 pandemics | Challenges of Cleft Surgeries in low- and middle-income countries (LMIC) during the COVID-19 pandemic | Asynchronous (MOOC); Synchronous (ZOOM) | T: Lectures & self-learning  P: Live Discussion with University Professors and Smile Train Experts; Virtual visit to Cleft Center Indonesia;  AA: Pre-test, post-test | Basic understanding of the challenges of cleft lip and palate surgical treatment in low middle-income countries during COVID-19 pandemics; Basic understanding of orthodontic treatment in cleft lip and palate patients | Post-test for each study material: Multiple choice questions | 18 |
|  |  |  | Impact of Covid-19 on Cleft Surgery in the Year 2020 |  |  |  |  |  |
|  |  | To identify the basic principles of orthodontic treatment in cleft lip and palate patients | Orthodontic Treatment |  |  |  |  |  |
|  |  |  | Orthognathic Surgery |  |  |  |  |  |
|  | 2 | To identify the importance of nutritional therapy in cleft lip and palate patients | Nutritional Therapy | Asynchronous (MOOC); Synchronous (ZOOM) | T: Lectures & self-learning  P: Live Discussion with University Professors and Smile Train Experts; Virtual visit to Cleft Center Indonesia;  AA: Pre-test, post-test | Basic understanding of nutritional therapy in cleft lip and palate patients; basic understanding of social care in cleft lip and palate patients | Assignment: Self-assessment for this week's learning activities  Post-test for each study material: Multiple choice questions | 18 |
|  |  |  | Cleft Nutrition |  |  |  |  |  |
|  |  | To identify the basic principles of social care in cleft lip and palate patients | Social Care for People with Orofacial Cleft |  |  |  |  |  |
|  | 3 | To identify the basic principles of cleft lip and palate’s impacts on speech and its management | Velopharyngeal Dysfunction | Asynchronous (MOOC); Synchronous (ZOOM) | T: Lectures & self-learning  P: Live Discussion with University Professors and Smile Train Experts; Virtual visit to Cleft Center Indonesia;  AA: Pre-test, post-test | Basic understanding of cleft lip and palate’s impacts on speech and its management; Basic understanding of the basic principles of multidisciplinary treatment in cleft lip and palate patients | Assignment: Self-assessment for this week's learning activities  Post-test for each study material: Multiple choice questions | 18 |
|  |  |  | The Impact of Cleft Palate on Speech |  |  |  |  |  |
|  |  |  | Cleft Speech Therapy in the Context of the Speech-Language Therapy Practice |  |  |  |  |  |
|  |  | To identify the basic principles of multidisciplinary treatment in cleft lip and palate patients | Management of New-Borns with Clefts During Covid-19 |  |  |  |  |  |
|  |  |  | Multidisciplinary Team Approach to Cleft Care |  |  |  |  |  |
|  |  |  | Telemedicine in Cleft Care |  |  |  |  |  |
|  | 4 | To identify the basic principles of outcome measures in cleft lip and palate treatment | Outcome Measures of Assessment for Oral Health and Dentofacial Aesthetics in Patients with Orofacial Clefts | Asynchronous (MOOC); Synchronous (ZOOM) | T: Lectures & self-learning  P: Live Discussion with University Professors and Smile Train Experts; Virtual visit to Cleft Center Indonesia;  AA: Pre-test, post-test | Basic understanding of outcome measures in cleft lip and palate treatment; basic understanding of cleft lip and palate epidemiology, prevention, and surveillance aspects | Assignment: final project  Post-test for each study material: Multiple choice questions | 18 |
|  |  |  | Standards for Reporting Outcomes of Cleft Care |  |  |  |  |  |
|  |  | To identity the basic principles of epidemiological aspect in cleft lip and palate | Epidemiology of Cleft Lip and Palate |  |  |  |  |  |
|  |  |  |  |  |  |  | **Total time (hour)** | **144** |

| **Description** | | **Assessment Category** | | **Learning outcome** |
| --- | --- | --- | --- | --- |
| **T** | **Tutorial** | Post-test for each topic | 80% | To understand and explain various aspects of cleft lip and palate |
| **P** | **Practicum** | Weekly assignments | 5% |  |
| **AA** | **Additional assignments** | Final project | 15% |  |
