# Supplementary material for: Utilising massive open online courses to enhance global learning dissemination in cleft lip and palate: a case report of penta helix collaboration
Source: BMC Med Educ. 2024 Mar 18;24:301. doi: 10.1186/s12909-024-05225-4 (PMC10949738; doi:10.1186/s12909-024-05225-4)
Supplement: Supplementary file 2 — Supplementary Material 2. [file 12909_2024_5225_MOESM2_ESM.docx]

**Additional file 2. The post-course survey questions and number of responses**

| 1. **Section I: course evaluation** | | | | | | | | | | | | | | | | | | | | | | | | | | | | | | | | |
| --- | --- | --- | --- | --- | --- | --- | --- | --- | --- | --- | --- | --- | --- | --- | --- | --- | --- | --- | --- | --- | --- | --- | --- | --- | --- | --- | --- | --- | --- | --- | --- | --- |
| **Please pick the most appropriate response**  **1: strongly disagree; 2: disagree; 3: neutral; 4: agree; 5: strongly agree** | | **Number of responses** | | | | | | | | | | | | | | | | | | | | | | | | | | | | | | |
|  |  | **1** | | | | **2** | | | | | | | **3** | | | | | | | **4** | | | | | | | **5** | | | | | |
| 1 | The course content as a whole was relevant | 0 | | | | 0 | | | | | | | 7 | | | | | | | 104 | | | | | | | 303 | | | | | |
|  | The course content as a whole was comprehensive | 0 | | | | 0 | | | | | | | 8 | | | | | | | 125 | | | | | | | 281 | | | | | |
|  | The course content as a whole was easy to understand | 0 | | | | 4 | | | | | | | 67 | | | | | | | 170 | | | | | | | 173 | | | | | |
| 2 | The course content pertaining to Cleft Lip and Palate was relevant | 1 | | | | 1 | | | | | | | 11 | | | | | | | 97 | | | | | | | 304 | | | | | |
|  | The course content pertaining to Cleft Lip and Palate was comprehensive | 0 | | | | 0 | | | | | | | 11 | | | | | | | 116 | | | | | | | 287 | | | | | |
|  | The course content pertaining to Cleft Lip and Palate was easy to understand | 0 | | | | 3 | | | | | | | 56 | | | | | | | 163 | | | | | | | 192 | | | | | |
| 3 | The course format: The ratio of lecture to Q&A content was appropriate | 0 | | | | 1 | | | | | | | 29 | | | | | | | 147 | | | | | | | 237 | | | | | |
|  | The course format: The format was conducive to learning | 0 | | | | 1 | | | | | | | 23 | | | | | | | 143 | | | | | | | 247 | | | | | |
|  | The Professors were knowledgeable | 0 | | | | 1 | | | | | | | 10 | | | | | | | 82 | | | | | | | 321 | | | | | |
| 4 | The Professors were: Well-prepared | 0 | | | | 1 | | | | | | | 16 | | | | | | | 102 | | | | | | | 295 | | | | | |
|  | The Professors were: Responsive to students' questions | 0 | | | | 2 | | | | | | | 16 | | | | | | | 113 | | | | | | | 283 | | | | | |
| 5 | What did you like most about this course, and why? | Open-ended questions | | | | | | | | | | | | | | | | | | | | | | | | | | | | | | |
| 6 | What did you like least about this course, and why? |  |  |  |  |  |  |  |  |  |  |  |  |  |  |  |  |  |  |  |  |  |  |  |  |  |  |  |  |  |  |  |
| 7 | Please provide up to three examples of how you will apply the learnings from this course: |  |  |  |  |  |  |  |  |  |  |  |  |  |  |  |  |  |  |  |  |  |  |  |  |  |  |  |  |  |  |  |
| **Please pick the most appropriate response**  **Yes/No** | | **Number of responses** | | | | | | | | | | | | | | | | | | | | | | | | | | | | | | |
|  |  | **Yes** | | | | | | | | | | | | | | | **No** | | | | | | | | | | | | | | | |
| 8 | Would you recommend this course to someone else? | 410 | | | | | | | | | | | | | | | 4 | | | | | | | | | | | | | | | |
|  | Why/why not | Open-ended questions | | | | | | | | | | | | | | | | | | | | | | | | | | | | | | |
| 1. **Section II: outcome evaluation** | | | | | | | | | | | | | | | | | | | | | | | | | | | | | | | | |
| **How would you rate…**  **Poor/Fair/Good/Excellent** | | **Number of responses** | | | | | | | | | | | | | | | | | | | | | | | | | | | | | | |
|  |  | **Poor** | | | | | | **Fair** | | | | | | | | | **Good** | | | | | | | | | **Excellent** | | | | | | |
| 9 | Your general knowledge and understanding of cleft lip and palate (CLP): a) Before course: | 160 | | | | | | 159 | | | | | | | | | 65 | | | | | | | | | 30 | | | | | | |
|  | Your general knowledge and understanding of cleft lip and palate (CLP): b) After course: | 1 | | | | | | 16 | | | | | | | | | 252 | | | | | | | | | 145 | | | | | | |
| 10 | Your general knowledge and understanding of how to properly treat CLP: a) Before course: | 211 | | | | | | 109 | | | | | | | | | 66 | | | | | | | | | 28 | | | | | | |
|  | Your general knowledge and understanding of how to properly treat CLP: b) After course: | 1 | | | | | | 33 | | | | | | | | | 257 | | | | | | | | | 123 | | | | | | |
| 11 | Your knowledge around the implications and challenges faced by CLP patients: a) Before course: | 173 | | | | | | 137 | | | | | | | | | 72 | | | | | | | | | 32 | | | | | | |
|  | Your knowledge around the implications and challenges faced by CLP patients: b) After course: | 2 | | | | | | 18 | | | | | | | | | 235 | | | | | | | | | 159 | | | | | | |
| **Please choose one or more**   1. **Genetics in Cleft** 2. **Presurgical Nasoalveolar Moulding** 3. **Rhinoplasty, Labioplasty, Palatoplasty, Velopharyngealplasty** 4. **Alveolar Bone Graft** 5. **Cleft Anaesthesia** 6. **Nutritional Therapy** 7. **Cleft Orthodontics** 8. **Cleft Speech Therapy** 9. **Multidisciplinary Team Approach to Cleft Care** 10. **Cleft Management During COVID-19** | | **Number of responses** | | | | | | | | | | | | | | | | | | | | | | | | | | | | | | |
|  |  | **Not selected** | **1** | | **2** | | | | **3** | | | **4** | | | **5** | | | **6** | | | | **7** | | **8** | | | | | **9** | | **10** | |
| 12 | What aspect of treating CLP did you learn the most about? | 0 | 59 | | 37 | | | | 56 | | | 7 | | | 6 | | | 72 | | | | 16 | | 45 | | | | | 73 | | 43 | |
| **Please choose one or more**   1. **Aetiology of Orofacial Cleft** 2. **Developmental Anatomy of CLP** 3. **Genetics in Cleft** 4. **Presurgical Nasoalveolar Moulding** 5. **Rhinoplasty, Labioplasty, Palatoplasty, Velopharyngealplasty** 6. **Alveolar Bone Graft** 7. **Cleft Anaesthesia** 8. **Nutritional Therapy** 9. **Cleft Orthodontics** 10. **Cleft Speech Therapy** 11. **Multidisciplinary Team Approach to Cleft Care** 12. **Cleft Management During COVID-19** 13. **Epidemiology of Cleft Lip and Cleft Palate** 14. **Telemedicine in Cleft Care** | | **Number of responses** | | | | | | | | | | | | | | | | | | | | | | | | | | | | | | |
|  |  | **Not selected** | **1** | **2** | | | **3** | | | **4** | **5** | | | **6** | | **7** | | | **8** | | **9** | | **10** | | 11 | | | 12 | | 13 | | 14 |
| 13 | From the topics of the course, which topic is your most favourite and would like to learn more about? | 0 | 27 | 13 | | | 20 | | | 21 | 56 | | | 12 | | 7 | | | 48 | | 37 | | 42 | | 48 | | | 21 | | 10 | | 52 |
| 14 | From the lectures offered, which topic is the easiest and most understandable? | 0 | 93 | 40 | | | 15 | | | 11 | 10 | | | 6 | | 3 | | | 58 | | 4 | | 19 | | 38 | | | 36 | | 9 | | 72 |
| 15 | What further training, or support would you need to improve your understanding of how to treat CLP patients? | Open-ended questions | | | | | | | | | | | | | | | | | | | | | | | | | | | | | | |
| 1. **Section III: general information and additional comments** | | | | | | | | | | | | | | | | | | | | | | | | | | | | | | | | |
| 16 | Please fill in the box with the name of your country | Open-ended questions | | | | | | | | | | | | | | | | | | | | | | | | | | | | | | |
| 17 | If others, please specify |  |  |  |  |  |  |  |  |  |  |  |  |  |  |  |  |  |  |  |  |  |  |  |  |  |  |  |  |  |  |  |
| 18 | Please fill in the box with your area of specialty |  |  |  |  |  |  |  |  |  |  |  |  |  |  |  |  |  |  |  |  |  |  |  |  |  |  |  |  |  |  |  |
|  | If others, please specify |  |  |  |  |  |  |  |  |  |  |  |  |  |  |  |  |  |  |  |  |  |  |  |  |  |  |  |  |  |  |  |
| 19 | Additional comments: |  |  |  |  |  |  |  |  |  |  |  |  |  |  |  |  |  |  |  |  |  |  |  |  |  |  |  |  |  |  |  |
